# Activation of Lymphocyte Cytolytic Machinery: Where are We?

**DOI:** 10.3389/fimmu.2013.00390

**Published:** 2013-11-19

**Authors:** Ricciarda Galandrini, Cristina Capuano, Angela Santoni

**Affiliations:** ^1^Department of Experimental Medicine, Istituto Pasteur-Fondazione Cenci-Bolognetti, Fondazione Eleonora Lorillard Spencer Cenci, Sapienza University, Rome, Italy; ^2^Department of Molecular Medicine, Istituto Pasteur-Fondazione Cenci-Bolognetti, Fondazione Eleonora Lorillard Spencer Cenci, Sapienza University, Rome, Italy

**Keywords:** NK cell, CTL, cytotoxicity, cytolytic synapse, signal transduction

## Abstract

Target cell recognition by cytotoxic lymphocytes implies the simultaneous engagement and clustering of adhesion and activating receptors followed by the activation of an array of signal transduction pathways. The cytotoxic immune synapse represents the highly specialized dynamic interface formed between the cytolytic effector and its target that allows temporal and spatial integration of signals responsible for a defined sequence of processes culminating with the polarized secretion of lytic granules. Over the last decades, much attention has been given to the molecular signals coupling receptor ligation to the activation of cytolytic machinery. Moreover, in the last 10 years the discovery of genetic defects affecting cytotoxic responses greatly boosted our knowledge on the molecular effectors involved in the regulation of discrete phases of cytotoxic process at post-receptor levels. More recently, the use of super resolution and total internal reflection fluorescence imaging technologies added new insights on the dynamic reorganization of receptor and signaling molecules at lytic synapse as well as on the relationship between granule dynamics and cytoskeleton remodeling. To date we have a solid knowledge of the molecular mechanisms governing granule movement and secretion, being not yet fully unraveled the machinery that couples early receptor signaling to the late stage of synapse remodeling and granule dynamics. Here we highlight recent advances in our understanding of the molecular mechanisms acting in the activation of cytolytic machinery, also discussing similarities and differences between Natural killer cells and cytotoxic CD8^+^ T cells.

## Introduction

Natural killer (NK) cells and cytotoxic T lymphocytes (CTLs) are major actors in immune protection against viral infections and cell transformation, and also mediate, in certain conditions, the killing of autologous or allogeneic un-diseased cells (1, 2). Target cell killing can occur upon the polarized secretion of cytotoxic mediators, such as perforin and granzymes, stored in specialized secretory lysosomes termed lytic granules (3).

While CTLs are activated by specific antigen recognition, the activation of NK cells is regulated by a balance of activating and inhibitory signals through a multitude of germ-line encoded receptors following the recognition of ligands expressed on the surface of target cells (4).

Based on recent acquisitions, this review attempts to draw a comprehensive picture on the coupling of receptor proximal signals to the late stages of synapse remodeling and granule dynamics; rather than covering how signals from discrete activation receptors cooperate to control NK-cell activation, a topic which has been extensively addressed in recent excellent reviews (5), we would try to recapitulate for every individual phase of the cytolytic process how the molecular signals arising upon receptor ligation are coupled to the distal molecular effectors responsible for the activation of cytolytic machinery, also highlighting the differences between CTLs and NK cells.

## Cytolytic Synapse Formation

The cytotoxic event is a well defined multistep process starting with the formation of a cell–cell contact specialized area called cytolytic synapse (3, 6) devoted to the polarized secretion of cytotoxic molecules.

Upon target recognition, receptors and signaling molecules rapidly segregate in the cytolytic synapse forming a supramolecular activation cluster (SMAC) that can be divided into concentrical zones: the central (cSMAC) and the peripheral (pSMAC) SMAC that is thought to be the focal point for the exocytosis of secretory lysosomes.

The formation of a mature synapse is not always essential for cell lysis by CTLs (7, 8), but it is believed to increase the efficiency of lytic granule polarization and target cell killing (9). Indeed, intra-vital imaging of the behavior of individual CTL or NK-cell infiltrating solid tumors in a mouse model has revealed that while CTLs tend to form more stable contacts with tumor cells, NK cells establish dynamic contacts (10).

An early stage in the commitment to cytolytic synapse formation is actin reorganization. As shown by 3-D confocal microscopy studies, actin rapidly polymerizes at the synapse periphery of both CTLs and NK cells to arrange a dense ring of cortical F-actin surrounding a central area through which lytic granules are secreted (6, 11).

Recently, the model of NK cells secreting lytic granules through a central region devoid of F-actin has been exceeded. A couple of companion papers (12, 13), both using very high-resolution imaging techniques, reveal that F-actin forms a pervasive network at the synapse, and that following activating receptor engagement, lytic granules are secreted through the filamentous network by accessing minimally sufficient sized clearances instead of a large-scale clearing of actin filaments. Such remodeling of cortical actin occurs within the central region of the synapse establishing secretory domain where lytic granules dock.

Strictly dependent on actin dynamics, activating signals are initiated by the formation of receptor micro-clusters at the periphery of the synapse in CTLs (14) and NK cells (15) undergoing a centripetal migration toward the synapse center. This movement is directed by actin depolymerization flow from an actin-rich periphery into an actin-poor area as shown by total internal reflection fluorescence microscopy (TIRF)-based studies in live T cells on lipid bilayer (16, 17).

**Figure 1:**
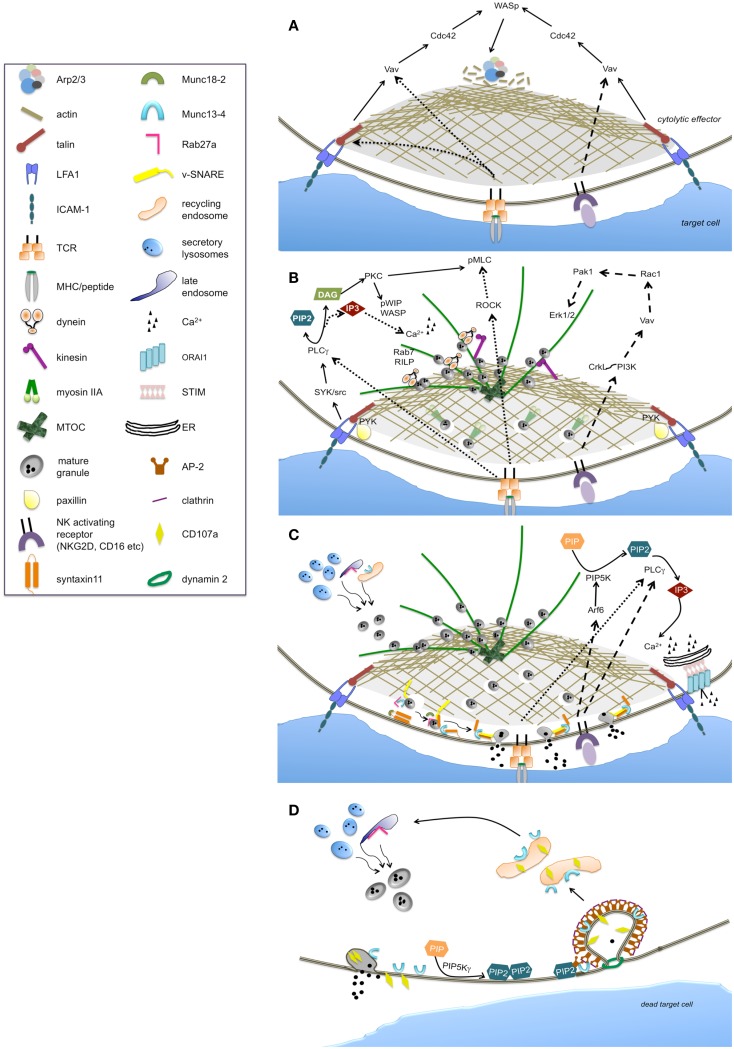
**Main signaling pathways and molecular effectors implicated in the regulation of individual phases of the cytolytic process**. The sequence of steps leading to the activation of cytolytic machinery are represented as: lytic synapse formation **(A)**, secretory apparatus polarization **(B)**, lytic granule secretion **(C)**, and lytic granule trafficking and retrieval **(D)**. The continuous line arrows indicate the signaling pathway likely common to NK cells and CTLs. Large-dashed arrows indicate the exclusive pathways of NK cells, while fine-dashed arrows indicate the exclusive pathways of CTLs.

Although, LFA1 ligation by ICAM-1 can signal on its own in NK cells (18), the formation of a stable and symmetric F-actin ring at cytolytic synapse requires integrin and NKG2D activating receptor co-ligation (12, 19). Similarly, in T cells, T cell receptor (TCR) and LFA1 co-aggregation is needed for the efficient synapse formation (20) (Figure 1A).

Downstream to LFA1, Cdc42 becomes active (21) and exhibits an oscillatory activation behavior at NK synapse (22); its molecular effector, Wiskott–Aldrich syndrome protein (WASp) is directly responsible for actin polymerization through the activation of the actin nucleator Arp2/3 complex. Accordingly, in the absence of WASp, as it occurs in the immune disorder WAS, or in the presence of actin inhibitors, F-actin accumulation at the synapse and the ability to kill is reduced in both NK cells (23–25) and CTLs (26) (Figure 1A). WASp activation strictly depends on phosphoinositide phosphatidylinositol 4,5-bisphosphate (PIP2) that is rapidly consumed at the cytolytic synapse (27). In this context the role of the actin binding protein talin has been clarified: its binding to the cytoplasmic tail of beta2 integrin mediates the recruitment of Arp2/3 which initiates actin polymerization upon LFA1 ligation (28).

## Secretory Apparatus Polarization

The activation of cytolytic machinery is achieved through a strong cell polarization driven by the reorganization of microtubule and actin cytoskeleton allowing the polarized secretion of lytic granules (Figure 1B).

Lytic granule journey starts with a retrograde minus-end transport on microtubules toward the centrosome or microtubule-organizing center (MTOC), followed by movement of the MTOC with clustered granules toward the edge of cSMAC both in CTLs and NK cells (29, 30). Microtubule-based molecular motor dynein has been implicated in the retrograde transport of granules to the MTOC and the subsequent movement of the MTOC toward the immune synapse in an actin-independent manner (31, 32). While in T cells the MTOC was believed to associate closely with the synapse to directly deliver lytic granule without the need of additional plus-ended granule motors (29), a recent report demonstrates that the microtubule motor protein kinesin-1 complexed to the small G protein Rab27a and synaptotagmin-like protein (slp)-3, acts in the terminal anterograde transport of cytotoxic granules close to the plasma membrane in CTLs (33). Whether kinesin-1 is required for the final microtubule transport to position lytic granule in NK cells needs further studies.

Recently, describing a spatio-temporal dissociation of the MTOC with lytic granules, the question of the requirement of MTOC polarization for efficient lethal hit delivery in CTLs has been raised (34). Beside microtubules, the movement of the MTOC/lytic granule complex toward the synapse also involves actin dynamics (6, 32). Indeed, in a pre-final step, the motor protein non-muscle myosin IIA mediates F-actin association with lytic granules (35, 36) and drives the final transit through the minimal clearance in the F-actin network across the cytolytic synapse, thus allowing granule approximation to synaptic membrane in NK cells. Furthermore, also in CTLs the two motor complexes dynein and myosin II has been described to work in a collaborative manner (37). The mutation of the gene encoding myosin IIA leads to May–Hegglin anomaly implying a reduced NK cytotoxicity (38).

In human NK cells signals for granule polarization can be uncoupled from degranulation: LFA1 engagement by ICAM-1 is sufficient to induce granule redistribution (18) thus featuring the minimal requirement for secretory lysosome polarization.

PLC gamma is regarded as a major factor in driving microtubule polarity and granule redistribution. Recently, LFA1-dependent Syk phosphorylation has been linked to the activation of PLC gamma-protein kinase C (PKC)-dependent pathway required for granule polarization (39) in NK cells. Additionally, Src kinases downstream to activating receptors, have been also implicated in the repositioning of MTOC and lytic granule both in CTL and NK cells (40, 41). Moreover, beta2 integrin-dependent phosphorylation of the molecular scaffold paxillin that associates with the tyrosine kinase Pyk2 has been shown to participate in directing MTOC polarization at NK synapse (42, 43). In CTLs the combined signal LFA1 and TCR is required for paxillin recruitment (44) to the site of integrin engagement.

PLC gamma defines a critical polarization pathway mediating the localized accumulation of second messenger diacylglycerol (DAG), which in turn promotes the recruitment of dynein at the MTOC/granule clusters (45). Notably, in agreement with the failure of integrin receptors to be coupled to Ca^2+^-dependent pathways, polarization resulted unaffected in the absence of calcium signaling in NK cells (39, 41). Differently in CTLs, variation in the Ca^2+^ concentration is thought to determine the kinetics of granule recruitment to the MTOC (9, 46).

Downstream to DAG, MTOC dynamics and polarization is driven by PKC isozymes recruited to the synaptic membrane. In CTLs, PKCη, PKCε, and PKCθ function redundantly to regulate the two motor complexes dynein and myosin II in driving MTOC polarization. Recent findings clarified how TCR signaling is coupled to the force-generating machinery demonstrating that PKCs activity controls myosin II localization directly by phosphorylating inhibitory sites within the myosin regulatory light chain; concurrently, Rho kinase (ROCK), mediating the phosphorylation of distinct sites within myosin regulatory light chain, induces myosin II clustering behind the MTOC (37). Additionally, PKC delta has been shown to co-localize with polarized granules in T cells (47).

In NK cells PKCθ is required for the WASp interacting protein WIP activation and association with secretory lysosomes at cytolytic synapse; the subsequent interaction with F-actin and myosin IIA (48, 49) links lytic granules to the actomyosin-dependent movement.

Another protein that plays a role in lytic granule movement in CTLs, is the small GTPase Rab7 acting by recruiting dynein-dynactin motor complex to secretory lysosomes through its molecular effector Rab interacting lysosomal protein, RILP, (50). Rab7 is recruited to the WASp-WIP-F-actin complex at the NK lytic synapse (48).

Cdc42-dependent signals have also been implicated in MTOC polarization through CDC42-interacting protein (CIP4), which contributes to anchor MTOC to the synapse by interacting with WASp and tubulin (51).

Proximal signals required for MTOC polarization in NK cells also include the extracellular regulated kinases, Erk1/2. The well-characterized phosphatidylinositol 3-kinase (PI3K)-dependent Rac1 → p21-activated kinase 1 (Pak1) → MEK-ERK1/2 pathway has been long referred as critical for lytic granule polarization in NK cells (43, 52, 53). On the opposite, in CTLs Erk activity is dispensable for MTOC reorientation (44). Erk activation has been uncoupled from integrin receptors while it is turned on downstream to activating receptors. Following NKG2D ligation for instance, PI3K-dependent pathway involving the adaptor CrkL and the small GTPase Rap1, has been shown to be required for MTOC polarization (54).

Despite multiple evidences, the involvement of Erk in microtubule remodeling is not fully understood. Erk2 was found to co-localize with microtubules (55), whereas paxillin phosphorylation has been linked to the PI3K-Erk pathway (56).

The final part of granule journey involves actin remodeling which occurs independently from LFA1 co-ligation (57), and defines a further “checkpoint” in the cytotoxic process: recent evidences demonstrate that NK-cell activation through several activating receptors including CD16 and NKG2D, leads to the remodeling of the cortical actin mesh at the synapse center to produce discrete nanometer-scale domains, as above mentioned (12). Contextually, micro-clusters of Grb2 and Vav1 signaling molecules rapidly reorganize to form a ring-shaped structure at the synapse center. Indeed, the pathway Vav1 → Rac1 → Pak1 is activated following CD16 and NKG2D ligation (58, 59) and regulates actin and microtubule dynamics (53) (Figure 1B). Whether remodeling of cortical actin within the central region of the NK-cell synapse is also controlled by this pathway, requires further investigation.

## Lytic Granule Secretion

Once lytic granules have reached the plasma membrane they are highly dynamic and mobile (60) and, contextually, docking and priming occur. Rab27a GTPase plays a critical role in granule docking; ultrastructural analysis evidenced that polarized granules fail to dock at the plasma membrane in response to TCR stimulation (61). The gene encoding Rab27a is mutated in the immunodeficiency Griscelli syndrome 2 (GS2) and in Ashen mice, resulting in severely reduced cytotoxic activity in CTLs (62, 63). In CTLs Rab27a binds to the synaptotagmin-like proteins Slp1 and Slp2 facilitating granule tethering (64, 65). In NK cells a Rab27a-independent pathway for CD16-mediated killing has also been reported (66). The finding of a residual degranulation in NK cells from GS2 patients is thought to indicate a relative redundancy of Rab protein activity (67).

Upon granule docking, the Rab27a binding partner, Munc13-4, mediates the priming of lytic granule in CTLs and NK cells (68, 69). Munc13-4 is mutated in familial hemophagocytic lymphohistiocytosis type 3 (FHL3); in the absence of Munc13-4, cytotoxic granules dock at the site of secretion but cannot fuse with the plasma membrane, thus leading to a severe reduction of cytotoxic activity. Munc13-4 has been postulated to open the conformation of the target (t-)SNARE syntaxin11 by removal of Munc18-2 which is required for syntaxin11 stabilization in NK cells (70). Indeed, Munc18-2 mutation causes FHL5 resulting in reduced granule exocytosis in NK cells (71). Moreover, mutations of syntaxin11 in FHL4 (72, 73) implicate a partial impairment of granule exocytosis both in NK cells and CTLs; the observation that the defect can be partially restored by IL-2 stimulation suggests the redundancy of syntaxin isoforms in the secretory mechanisms. Functional studies have shown that deficiency in vesicle (v-)SNAREs, VAMP7, VAMP8, and VAMP4 results in defective granule exocytosis (74–76) (Figure 1C).

An additional role of Munc13-4 in enabling the maturation of perforin-containing granules has been highlighted in CTLs: a seminal study demonstrated that close to the cytolytic synapse, Munc13-4 promotes the coalescence of a pool of endosomal/recycling vesicles carrying effectors of cytolytic machinery such as Rab27a and Munc13-4, with perforin-containing granules, leading to the formation of a unique mature “exocytic vesicle” (77). The observation that in NK cells neither Rab27 or Munc13-4 are present on the lytic granule surface but associate with them upon receptor engagement, suggests a similar role; moreover, separate signaling routes are used in NK cells to direct Munc13-4 and Rab27a to lytic granule (67).

While the nature of the signals that couples activating receptors to secretory machine remains partially unknown, the strict calcium-dependence remains the hallmark for lytic granule secretion (78). The essential role of PLCgamma2 in granule exocytosis has been demonstrated in knock out mice (79). Downstream to TCR or NK activating receptors, the activation of PLC gamma results in the hydrolysis of PIP2 to generate the second messenger inositol-1,4,5-trisphosphate (IP3) which triggers the mobilization of intracellular Ca^2+^ from endoplasmic reticulum; the resulting depletion of Ca^2+^ stores and aggregation of endoplasmic reticulum Ca^2+^ sensor, STIM, trans-activate the plasma membrane Ca^2+^ release-activated Ca^2+^ channel ORAI1, leading to the store operated Ca^2+^ entry (SOCE). Patients with mutation in either STIM1 or ORAI1 exhibit a defect in secretion, whereas lytic granule polarization results unaffected (80). Phosphatidylinositol-4-phosphate 5-kinase (PIP5K)alpha and gamma isoforms are the enzymes mainly responsible for PIP2 synthesis in NK cells; they synergistically act in the control IP3/Ca^2+^ levels required for lytic granule exocytosis (27); in contrast, PIP5Ks isoenzymes behave redundantly in the control of granule polarization which is in line with the lack of Ca^2+^-dependence, as above mentioned. Downstream to CD16, PIP5Kalpha activation is regulated by small G protein Arf6; accordingly, interfering with Arf6 function leads to reduced lytic granule exocytosis (81).

The Ca^2+^-dependent factors required for lytic granule exocytosis are largely unknown. The high-affinity Ca^2+^ binding protein synaptotagmin is a possible candidate; recently, synaptotagmin VII has been implicated in exocytosis of lytic granules (82) (Figure 1C).

Because Munc13-4 contains two C2, Ca^2+^ binding domains, it also might represent a Ca^2+^ sensor for exocytosis. Actually, the translocation of Munc13-4 to membrane rafts, indicating granule fusion with plasma membrane, occurs in Ca^2+^-independent manner (83).

## Lytic Granule Trafficking and Retrieval

The demonstration that Rab11+ recycling endosome polarizes at cytolytic synapse along with secretory apparatus in order to allow Munc13-4-dependent granule maturation into a fusion-competent lytic organelle (77), have raised the disrupting concept that the cytolytic synapse behaves as a focal point for both exocytosis and endocytosis. Indeed, a bidirectional trafficking of lytic granule proteins exposed at the plasma membrane on degranulation has been demonstrated; both lysosome-associated membrane protein-1 (LAMP-1, also known as CD107a) (84) and Munc13-4 (83) undergo a rapid endocytosis, leading to the hypothesis that granule exocytosis is coordinated with the retrieval of cytolytic machinery components. Additionally, cytolytic mediators are also recaptured into early endosomes of NK cells via a clathrin-dependent route after target cell stimulation thus contributing to the cytolytic potential (85).

In this framework, the ability of cytolytic effectors to execute multiple killing cycles in a short time period (86–88) is thought to depend both on the release of a fraction of lytic granules (77) and on a continuum refilling of the granule store through newly synthesized cytotoxic mediators (89). Whether secretory lysosome retrieval could facilitate recycling and reusing of cytotoxic machinery components thus contributing to the serial killing potential, is not fully understood.

The molecular signals controlling the endocytic traffic at cytolytic synapse have begun to be clarified. Recent findings reported that a constitutive PIP5Kgamma-dependent PIP2 pool is involved in the control of Munc13-4 re-internalization through a clathrin/AP2 dependent endocytic route, which is functional to ensure the full serial killing potential in NK cells (83). Such findings strengthen the analogy between neuronal and cytolytic synapse where PIP5K gamma also triggers the clathrin-mediated retrieval of synaptic vesicles (90) (Figure 1D).

An additional effector molecule involved in granule recapture during exocytosis can be the fission factor dynamin 2 which have been shown to be required for cytotoxicity in NK cells (91).

## Perspectives

One of the aspects that remains enigmatic in the biology of lymphocyte cytotoxicity is the serial killing potential. The ability of NK cells and CTLs to mediate the sequential attack of successive targets by a single effector was suggested in early observations; only recently, however, intra-vital and real-time imaging of a single cell behavior have shed light on a marked heterogeneity in the cytotoxic potential and on relevant differences in the contact dynamic with target cells (87, 88): CTLs forming stable independent synapse are able to simultaneously bind and attack multiple targets, while NK cells forming short-lived synapse, allows the detachment and the subsequent engagement of other targets. Intriguingly, NK cells are proposed to integrate signals arising form previous and current targets resulting in a continuous signaling that persist until formation of a new synapse (87). A considerable challenge for the future will be the understanding of the spatial and temporal coordination of molecular signals which may account for different qualities of cytolytic responses. In such framework the knowledge on how NK-cell contact with target is terminated and the molecular basis of retrieval processes also represent future challenges.

Also we need to learn more on NK-cell education: in particular, while the molecular basis of MHC-I-dependent licensing have began to be clarified (92, 93), completely unknown remains the molecular basis of NK hypo-responsiveness that follows the sustained stimulation of certain activating receptors.

The unraveling of NK functional plasticity would have a major impact in NK-cell-based immunotherapeutic approaches and could drive a renewed interest in signal transduction processes.

## Conflict of Interest Statement

The authors declare that the research was conducted in the absence of any commercial or financial relationships that could be construed as a potential conflict of interest.
